# Essential Trace and Toxic Element Content in Lacaune Sheep Milk during Lactation

**DOI:** 10.3390/foods12234291

**Published:** 2023-11-28

**Authors:** Zvonko Antunović, Boro Mioč, Josip Novoselec, Ivan Širić, Valentino Držaić, Željka Klir Šalavardić

**Affiliations:** 1 Department for Animal Production and Biotechnology, Faculty of Agrobiotechnical Sciences Osijek, Josip Juraj Strossmayer University of Osijek, 31000 Osijek, Croatia; zantunovic@fazos.hr (Z.A.); zklir@fazos.hr (Ž.K.Š.); 2 Department of Animal Science and Technology, Faculty of Agriculture, University of Zagreb, 10000 Zagreb, Croatia; bmioc@agr.hr (B.M.); isiric@agr.hr (I.Š.); vdrzaic@agr.hr (V.D.)

**Keywords:** sheep, Lacaune, lactation, milk, essential and toxic elements

## Abstract

The aim of this study was to investigate the concentrations of essential trace and toxic elements in the milk of Lacaune sheep during lactation in intensive rearing systems. This research was conducted with 30 Lacaune sheep that were monitored in the early (60 days of lactation), medium (120 days of lactation), and late (180 days of lactation) stages of lactation. The sheep were fed a pelleted feed mixture (1 kg/day), a cereal mixture (600 g/day), and alfalfa hay (ad libitum). The essential (Fe, Zn, Cu, Co, Mn, Mo, Se, Cr, and Ni) and toxic element (heavy metals: Cd, Pb, As, and Hg) concentrations in the feed and milk were determined using an inductively coupled plasma mass spectrometer. Significant variations in the main essential trace and toxic elements, except for the Mo, Se, Ni, As, and Hg concentrations, were found in the milk of Lacaune sheep during lactation. As lactation progressed, in the late stage of lactation, significantly higher concentrations of Co, Mn, Mo, Cr, and Pb were found, while Zn and Cu in the milk of Lacaune sheep decreased significantly (4.15 and 0.21 mg/kg) compared to their concentrations in the early stage of lactation (5.66 and 0.43 mg/kg). Significantly lower concentrations of Fe and higher concentrations of Cd were found in the medium stage (0.23 mg/kg and 1.08 µg/kg) of lactation compared to both the early and late stages of lactation. An analysis of the correlation coefficients between the essential trace and toxic elements in Lacaune sheep milk during lactation determined a significantly positive correlation between Fe:Cr, Fe:Mn, Fe:Co, Fe:Se, Zn:Ni, Zn:Se, Cr:Mn, Cr:Co, Cr:Se, Cr:Mo, Mn:Co, Mn:Pb, Co:Ni, Co:Se, Ni:Se, Se:Mo, Se:Pb, and Cd:Pb. A significantly negative correlation was also found between Cu:Mn, Zn:Mo, Cg:Hg, and Hg:Pb. Based on the obtained results, it is recommended that the influence of the stage of lactation, as well as the breed of sheep, should be included when designing experiments. In general, sheep milk is rich in essential trace elements, but it also contains a very low content of toxic elements, which provides justification for increasing the breeding of Lacaune sheep and indicates the convenience of consuming their milk without risking the consumer’s health.

## 1. Introduction

Milk and dairy products constitute an important source of energy and nutrients for humans [1]. Sheep milk has an increasingly important role in the nutrition of the human population in the world. Its value is emphasized by an up to 2–3 times higher cost price compared to cow’s milk, which significantly affects the increase in the breeding of dairy sheep breeds as well as the economy of production [2,3]. The above is followed by extensive comprehensive research on the quality of sheep milk through the determination of the content of essential trace and toxic elements. Numerous studies have been conducted on this topic, in which significant effects of various factors, primarily feeding, the breeding system, the stage of lactation, the parity, and other factors, were determined [4,5,6,7,8,9]. However, the aforementioned studies were conducted with a larger number of sheep breeds, while in the available literature, there is a very small number of studies on and determined concentrations of these trace and toxic elements in the milk of Lacaune sheep [8,10]. Knowing the quality of sheep milk by analyzing the content of essential trace and toxic elements in the milk is very important. The reason for the above is not only the influence on the growth and development of suckling lambs but also the fact that the majority of sheep milk is used to make cheese, which is an important food in the human diet. Milk and its products are sources of essential macro- and micronutrients, thus presenting a large source of nutrients, especially for children [11]. Milk contains significant amounts of essential trace elements (Zn, Co, Mo, Se, Cr, and Ni), but also very small amounts of toxic elements (heavy metals: Cd, Pb, As, and Hg) [4,7,12]. Trace elements such as iron (Fe), zinc (Zn), copper (Cu), cobalt (Co), manganese (Mn), molybdenum (Mo), selenium (Se), chrome (Cr), and nickel (Ni) are essential, with biochemical and physiological roles in organisms [13]. These depend on each specific element; for example, Cu and Fe are involved in homeostasis, Zn has a role in many enzymatic and biochemical reactions, and Mn is involved in the function of the immune system, homeostasis, and coagulation [14]. However, if certain threshold concentrations of these elements are elevated, a homeostatic disturbance may occur, with bioaccumulation in the body. In this case, free radicals can be formed, leading to oxidative stress that may harm human health [15,16]. Toxic elements (Pb, Cd, As, and Hg) have no recorded physiological functions in the body, and even at low concentrations, they can be toxic to the body and cause numerous disorders, especially with prolonged exposure (physical and psychological disorders, but also neurodegenerative diseases and the appearance of cancer) [17,18]. Heavy metals may be transferred to milk from milk containers, during processing, or through contaminated water, livestock feed, or the environment [19,20]. The influence of the stage of lactation on the concentrations of essential trace and toxic elements in milk has not yet been fully investigated in Lacaune sheep under intensive rearing conditions. Therefore, the aim of this study was to investigate the concentrations of essential trace and toxic elements in the milk of Lacaune sheep during lactation in intensive rearing systems.

## 2. Materials and Methods

### 2.1. Design of the Experiment and Animal Selection

This research was conducted with Lacaune sheep at the Orkić family farm in Gundinci (latitude: 45.155; longitude: 18.492). The selection of sheep was carried out immediately after the suckling period from a herd of 200 sheep, with an emphasis on good health and physical condition, as well as a uniform age, stage of lactation, and number of lambs in the litter. A group of 30 sheep was formed with an average live body weight of 61 kg, and the sheep were monitored in the early (60 days ± 5 days), medium (120 days ± 5 days), and late (180 days ± 5 days) stages of lactation.

This research was carried out by obeying the legal provisions determined by the Animal Protection Act (Republic of Croatia Official Gazette No. 133 (2006), No. 37 (2013), and No. 125 (2013)), and approved by the Committee for Animal Welfare of the Faculty of Agrobiotechnical Sciences.

### 2.2. Collection and Analysis of Feed and Milk Samples

The sheep were fed with diets based on a pelleted feed mixture (15% crude protein) in the amount of 1 kg/day, a cereal mixture (oats and barley) in the amount of 600 g/day, and alfalfa hay (ad libitum). No refusals of feed mixtures were observed during the experiment. The water and salt were offered ad libitum. Three feed samples of each type of feed were sampled in each stage of lactation and pooled to obtain one sample of the feed mixture, the cereal mixture, and the hay, which were subjected to analyses.

Milk was sampled from sheep in the early (60th day), medium (120th day), and late (180th day) stages of lactation during machine milking. Milk sampling was carried out during the morning milking in bottles (200 mL), individually from each ewe. After that, it was homogenized with a vortex, stored at 4 °C, and then transferred in a freezer at −80 °C.

Afterwards, the samples of milk and feed were prepared for digestion. Firstly, sampling bottles were sunk in 20% HNO_3_ for 24 h and then washed with deionized water in order to avoid contamination. According to the method described in Belete et al. [21], the digestion of feed and milk samples of sheep was conducted. Milk powder as a reference material (NCSZC73015, National Analysis Center for Iron and Steel, Beijing, China) was used for validation of the method. After that, 3.0 mL of the milk sample was transferred into a digestion vessel (60 mL); then, 6 mL of nitric acid (70%) and 1 mL of hydrogen peroxide (30%) were added. This mixture was shaken and left for 10 min. Samples of milk and feed were digested at the optimized microwave digestion program designed as follows: 50 W, 165 °C (10 min); 80 W, 190 °C (20 min); and 0 W, 50 °C (10 min). Digestion was carried out with a microwave system (Mars 6, CEM, Matthews, NC, USA). Afterwards, dilution of the digest was carried out to 25 mL with deionized water. Samples were then analyzed by using continuous flow hydride generation technique using inductively coupled plasma mass spectrometer (ICP-MS, Agilent 7500a, Agilent Technologies Inc., Santa Clara, CA, USA). Samples were analyzed for essential trace elements (Fe, Zn, Cu, Co, Mn, Mo, Se, Cr and Ni) and toxic elements (heavy metal: Cd, Pb, As and Hg). According to Bosnak et al. [22], digested milk samples provided for analyses of As were subjected to the pre-reduction step. For the pre-reduction of As, 20 mL of the sample was placed in a polypropylene autosampler tube (50 mL) and mixed with 2 mL of a KI and ascorbic acid solution (5%). A total of 6 mL of concentrated HCl was added in the mixture, which was left for at least 20 min. The sample was ready to run on ICP-MS, after it was brought to the 50 mL mark with deionized water. For the Hg and Se pre-reduction, a sample (20 mL) was placed in a 125 mL beaker, while concentrated HCl (20 mL) was added slowly. This solution was transferred to a polypropylene autosampler tube (50 mL) and diluted with deionized water to 50 mL. Samples were analyzed with ICP (Optima 21000 DV, Perkin Elmer, Waltham, MA, USA). Instrumental limits of detection and quantification for essential trace and toxic or heavy metal elements analyses in sheep milk and feed are presented in Table 1.

The content of essential trace elements, like Fe, Zn, Cu, Co, Mn, Mo, Se, Cr and Ni, and toxic elements or heavy metals, like Cd, Pb, As and Hg in the feed and water of Lacaune sheep during lactation are presented in Table 2.

### 2.3. Statistical Analyses

Obtained research results were processed by the MEANS procedure, while the influence of lactation stages was determined by the GLM procedure and processed by SAS 9.4^®^. Tukey’s test was used to determine comparison between mean values, and the differences between the groups were declared as significant at *p* < 0.05. Correlations among parameters of ewe milk were evaluated by Pearsons’ correlation using PROC, and correlations were significant only if *p* < 0.05.

## 3. Results

Table 3 presents descriptive statistics of essential trace and toxic elements in Lacaune sheep milk during lactation.

The analysis of average values of the determined concentrations of essential trace and toxic elements (Table 3) presented a significant deviation, especially for the concentrations of toxic elements (high standard deviation).

Significant variations were found for most of essential trace elements, except for the concentrations of Se and Ni in the milk of Lacaune sheep during lactation (Table 4). As lactation progressed, in the late stage of lactation, significantly higher concentrations of Co, Mn, Mo and Cr were found, while the concentrations of Zn and Cu in the milk of Lacaune sheep decreased significantly compared to the early stage of lactation. In the medium stage of lactation, significantly lower concentrations of Fe were determined in comparison to both the early and late stages of lactation, while a non-significant increase in Se concentrations and a decrease in Ni concentrations were observed in the milk of Lacaune sheep as lactation progressed. Toxic element (heavy metal) concentrations, primarily those of Cd and Pb, varied significantly during lactation, while no significant variations were recorded for the concentrations of As and Hg (Table 4) as concentrations were insignificantly increased and Hg concentrations decreased during the progress of lactation in the milk of Lacaune sheep.

By analyzing the correlation coefficients between essential trace and toxic elements in Lacaune sheep milk during lactation (Table 5), a significantly positive correlation was determined between Fe:Cr, Fe:Mn, Fe:Co, Fe:Se, Zn:Ni, Zn:Se, Cr: Mn, Cr:Co, Cr:Se, Cr:Mo, Mn:Co, Mn:Pb, Co:Ni, Co:Se, Ni:Se, Se:Mo, Se:Pb and Cd:Pb. A significantly negative correlation was also found between Cu:Mn, Zn:Mo, Cd:Hg and Hg:Pb.

## 4. Discussion

Sheep milk is a good source of some essential trace elements, especially Zn and Fe, and compared to other types of milk produced from domestic animals, it has an advantage in terms of the contents of milk [23]. However, the effect of sheep breed on essential trace and toxic elements in milk is very important in addition to the effects of feeding, lactation stage, parity and breeding system, [24]. Nutrition is a complex factor which is interdependent with numerous other factors; therefore, it is difficult to distinguish their mutual effects [25]. In the available literature, there is a very small number of studies, especially those examining each essential and toxic element in milk, conducted with Lacaune sheep [8,10]. In the present study, significant variations in the concentrations of essential trace elements, except for Se and Ni, were determined in the milk of Lacaune sheep during lactation (Table 5). As lactation progressed, in the late stage of lactation, significantly higher concentrations of Co, Mn, Mo and Cr were found, while Zn and Cu in the milk of Lacaune sheep decreased significantly compared to their contents in the early stage. Significantly lower concentrations of Fe were found in the medium stage compared to both the early and late stages of lactation.

Compared to the present study, Panayotov et al. [10] found similar average concentrations of Zn, and higher Fe and Cu (0.50, 0.10 and 0.04 mg/100 g) in the milk of Lacaune sheep in the first lactation in Bulgaria. Michlova et al. [8] found higher average concentrations of Zn in the milk of Lacaune sheep grazing in the Czech Republic at both studied locations, 20.33 (16.57–24.33 mg/kg) and 25.86 (17.71–34.27 mg/kg), and Cu only at one location (1.10 and 0.25 mg/kg) compared to the present study. Pšenkova et al. [26] in Slovakia found similar concentrations of Se (0.04 µg/kg), higher Fe (0.66 mg/kg), and lower Zn (4.10 µg/kg), while Cu concentrations were <0.50, Cd < 0.0040; As < 0.030, Hg < 0.002, Ni < 0.10 and Pb < 0.10 mg/kg in sheep milk because they were below the detection limit of the device. In the milk of Dubrovnik sheep, Antunović et al. [5] found similar changes in concentration of essential trace and toxic elements. These authors found lower Zn, Cu, Mn, Se, Cr and As, as well as higher Fe, Co, Cd and Pb concentrations. Guiso et al. [9] determined a lower average concentration of Cu (180 µg/kg) and higher Mn (100 µg/kg), Se (80 µg/kg), Zn (6300 µg/kg) and Ni (50 µg/kg).

Concentrations of trace elements in animal products are mostly related to animal feed. In conventional farming and intensive rearing in which the present study occurred, the major source of trace elements is mineral supplement which is routinely added to the concentrate mixture to ensure the animals’ physiological needs [27]. The decrease in Zn concentration in milk during lactation may be associated with zinc because of its high excretion in milk which is, along with its products, one of the major sources of dietary zinc [28].

As lactation progressed, Antunović et al. [5] found a significant decrease in Zn concentrations and an increase in Cu, Mn, Mo and Co in the milk of Dubrovnik sheep. In the research by Antunović et al. [6], a significant decrease in the concentration of most essential trace elements was found (Fe, Cu, Zn, Mn, Mo, Cr, Ni and Se) as lactation progressed (from 40 to 120 days of lactation). In the research by Pšenkova et al. [29], a significant decrease in Zn concentrations was detected from early (60 days), medium (90 days) to late stages of lactation (120 days), from 8.9 to 5.7 to 3.1 mg/kg of milk from the Tsigai breed, similar to the present study. The authors pointed out that the reduction in the concentration of elements in milk of sheep in lactation may be due to a decrease in mammary gland osmotic pressure during the hot summer temperatures. Water is known to play an important role in the processes involved in the formation of milk, since milk is isosmotic with plasma, while lactose allows the drainage of water from blood to the alveolar compartment, regulating the volume of milk production [30]. In the milk of Bulgarian dairy sheep, Ivanova et al. [31] determined higher concentrations of Fe and Se (0.8–1.3 and 25.5–17.7 mg/kg) and lower Zn (3.81–4.41 mg/kg) compared to the present study.

As in the present study, Pšenkova et al. [26] found a significant increase in Fe concentrations (from 0.59 to 0.81 mg/kg) and a decrease in Zn concentrations during lactation (from 4.90 to 3.40 mg/kg), but also a significant increase in Se concentrations as lactation progressed (from 0.031 to 0.063 mg/kg) in the milk of sheep reared in areas with environmental burden in Slovakia. Another research in Slovakia carried out by Almášiová et al. [32] showed higher concentrations of Fe and As (1.57 ± 0.28 and 0.06 ± 0.08 mg/kg) and lower Zn and Se (4.19 ± 0.46 and 0.19 ± 0.14 mg /kg) in sheep milk, in the region with potentially undisturbed area, compared to the present study, which is also indicated by its representation in diet and water (Table 2).

The concentrations of toxic elements (heavy metals), primarily Cd and Pb, varied significantly during lactation, while no significant variations were recorded for the concentrations of As and Hg (Table 4). Guiso et al. [9] determined a lower concentration of Cd (0.3 µg/kg) and higher Pb (13 µg/kg) in sheep milk in Italy. Accumulation of heavy metals (Cd and Pb) in ruminants is mostly related to feed grown on soil contaminated with these toxic elements [33]. However, in the present study, high values of these toxic elements in feed and water aimed for sheep were not determined as presented in Table 2. Antunović et al. [4] determined the average concentration of heavy metals (Cd = 0.006; Pb = 0.026, Hg = 0.021; As = 0.029 mg/kg DM milk) in the milk of Merinolandschaf sheep during lactation in early lactation (60 days after lambing). In the milk of Dubrovnik sheep, Antunović et al. [5] found lower As and higher Cd and Pb concentrations. In the milk of Travnička pramenka, Antunović et al. [6] found similar variations for the concentrations of As, Cd and Hg compared to the present study. Houpert et al. [34] pointed out that toxic metal content in animal organisms and milk may increase very fast, but its excretion through milk is very low. The increase in Cd concentration during lactation in the milk of Lacaune sheep is associated with an increase in the protein content of the milk as the lactation comes to an end. It is known that the yield of milk in sheep decreases as the lactation comes to an end, but the protein content in milk increases which is specific for the normal lactation curve in lactating sheep. In support of this is the fact highlighted by Mata et al. [35] that Cd is mainly related to the protein fraction (casein) obtained by coagulation. The increase in the concentration of Pb in the milk of Lacaune sheep can be the result of more Pb excreted via milk in comparison with that of cows. The determined concentrations of toxic elements are very low. Thus, it can be concluded that this milk is not a source of these toxic elements.

By analyzing the correlation coefficients between essential trace and toxic elements in Lacaune sheep milk during lactation (Table 5), numerous correlations were determined. In this study, a significantly positive correlation was found between Fe:Cr, Fe:Mn, Fe:Co, Fe:Se, Zn:Ni, Zn:Se, Cr:Mn, Cr:Co, Cr:Se, Cr:Mo, Mn:Co, Mn:Pb, Co:Ni, Co:Se, Ni:Se, Se:Mo, Se:Pb and Cd:Pb in Lacaune sheep milk during lactation. A significantly negative correlation was found between Cu:Mn, Zn:Mo, Cg:Hg and Hg:Pb. Pšenkova et al. [29] found a strong positive correlation between Fe:Se (0.985 **) in the milk of the Cigaya breed in early lactation, while Miedico et al. [36] determined a significant positive correlation in sheep milk between Fe and Mn (0.65; *p* < 0.01). In the milk of Dubrovnik sheep, Antunović et al. [5] found a strong positive correlation between Cr and Se (0.55; *p* < 0.01) and Cd and Pb (0.80; *p* <0.001). All of these correlations were expected due to their metabolic interrelations.

## 5. Conclusions

Based on the determined changes in essential trace and toxic elements concentrations in the milk of Lacaune sheep, we can conclude that when setting up the design of the experiment, the effect of the lactation stage should be included in the model. In addition, sheep milk is rich in essential trace elements, while a very low concentration of toxic elements is found. The abovementioned provides justification for increasing the breeding of Lacaune sheep and indicates the convenience of consuming their milk without risking the consumer’s health.

## Figures and Tables

**Table 1:** Instrumental limits of detection and quantification for the determination of essential trace and toxic elements in sheep milk and feed (mg/kg).

Parameters	Instrumental Limits of Detection	Instrumental Limits of Quantification
Fe	0.00073	0.00243
Zn	0.00143	0.00478
Cu	0.00322	0.01073
Co	0.0112	0.03733
Mn	0.03967	0.13222
Mo	0.04005	0.13349
Se	1.31133	4.37111
Cr	0.57387	1.91289
Ni	0.28553	0.95178
Cd	0.04344	0.1448
Pb	0.01147	0.03822
As	0.438	1.46
Hg	0.01216	0.04053

**Table 2:** Essential trace and toxic element content in Lacaune sheep feed.

Parameters	Feed			Water
	Feed Mixture	Cereal Mixture	Hay	
Fe, mg/kg DM	377.7	129.9	91.96	0.0059
Zn, mg/kg DM	165.80	20.93	9.58	0.0021
Cu, mg/kg DM	24.50	4.66	5.48	0.00082
Co, µg/kg DM	196.17	37.98	44.20	0.048
Mn, µg/kg DM	80,420	12,970	6038	27.98
Mo, µg/kg DM	725	452.7	1282	0.21
Se, µg/kg DM	225	41.24	15.29	4.60
Cr, µg/kg DM	1911	347.4	481.6	12.11
Ni, µg/kg DM	4045	890.8	1056	0.0435
Cd, µg/kg DM	196.5	55.69	201	0.0002
Pb, µg/kg DM	448.2	168.1	102.2	<LD
As, µg/kg DM	359.7	32.12	35.02	1.069
Hg, µg/kg DM	0.506	<LD	0.35	0.0057

DM—dry matter; LD—instrumental limit detection.

**Table 3:** Essential trace and toxic element descriptive statistics in Lacaune sheep milk during lactation.

Parameter, mg/kg	Mean	SD	Minimum	Maximum
Fe, mg/kg	0.33	0.18	0.05	1.05
Zn, mg/kg	4.90	1.34	2.00	8.75
Cu, mg/kg	0.29	0.16	0.09	0.95
Co, µg/kg	0.89	0.15	0.52	1.35
Mn, µg/kg	56.28	19.24	16.93	135.00
Mo, µg/kg	28.60	8.33	14.21	56.32
Se, µg/kg	34.32	5.75	24.67	53.75
Cr, µg/kg	78.59	19.65	42.45	133.50
Ni, µg/kg	7.72	1.29	5.42	14.65
Cd, µg/kg	0.60	0.53	0.02	2.15
Pb, µg/kg	0.91	0.73	0.21	4.23
As, µg/kg	1.29	0.34	0.54	2.12
Hg, µg/kg	0.10	0.17	0.0007	1.06

SD—standard deviation.

**Table 4:** Lactation stage effect on essential trace and toxic elements in Lacaune sheep milk.

Parameter	Stage of Lactation			SEM	*p*-Value
	Early	Medium	Late		
Fe, mg/kg	0.34 ^a^	0.23 ^b^	0.43 ^a^	0.019	<0.001
Zn, mg/kg	5.66 ^a^	4.84 ^b^	4.15 ^b^	0.141	<0.001
Cu, mg/kg	0.43 ^a^	0.23 ^b^	0.21 ^b^	0.017	<0.001
Co, µg/kg	0.83 ^b^	0.87 ^b^	0.97 ^a^	0.015	<0.001
Mn, µg/kg	42.19 ^c^	58.51 ^b^	69.26 ^a^	2.016	<0.001
Mo, µg/kg	25.25 ^b^	29.00 ^ab^	31.74 ^a^	0.873	0.009
Se, µg/kg	32.95	34.05	36.07	0.603	0.103
Cr, µg/kg	66.53 ^b^	69.30 ^b^	101.43 ^a^	2.060	<0.001
Ni, µg/kg	7.81	7.89	7.46	0.135	0.390
Cd, µg/kg	0.38 ^b^	1.08 ^a^	0.33 ^b^	0.055	<0.001
Pb, µg/kg	0.43 ^c^	1.41 ^a^	0.88 ^b^	0.077	<0.001
As, µg/kg	1.22	1.32	1.35	0.035	0.270
Hg, µg/kg	0.14	0.10	0.07	0.018	0.257

SEM—standard error of mean; ^a,b^—with different superscript letters differ significantly (*p* < 0.05).

**Table 5:** Correlation coefficient between essential trace and toxic elements in Lacaune sheep milk during lactation.

	Cu	Fe	Zn	Cr	Mn	Co	Ni	As	Se	Mo	Cd	Hg	Pb
Cu	1.00												
Fe	0.09 0.411	1.00											
Zn	0.25 0.019	−0.08 0.439	1.00										
Cr	−0.21 0.052	0.42 <0.001	−0.16 0.127	1.00									
Mn	−0.38 <0.001	0.30 0.003	−0.006 0.956	0.57 <0.001	1.00								
Co	0.03 0.76	0.46 <0.001	0.04 0.698	0.65 <0.001	0.41 <0.001	1.00							
Ni	0.33 0.001	0.21 0.041	0.06 0.549	0.12 0.256	0.005 0.965	0.44 <0.001	1.00						
As	−0.02 0.836	0.27 0.010	−0.09 0.371	0.16 0.136	0.22 0.034	0.26 0.011	0.19 0.076	1.00					
Se	0.17 0.105	0.33 0.001	0.09 0.410	0.55 <0.001	0.26 0.013	0.63 <0.001	0.42 <0.001	0.24 0.019	1.00				
Mo	−0.10 0.335	0.15 0.143	−0.32 0.002	0.40 <0.001	0.15 0.170	0.26 0.014	0.19 0.072	0.16 0.123	0.32 0.002	1.00			
Cd	−0.06 0.569	−0.240 0.022	−0.06 0.553	−0.13 0.220	0.07 0.491	0.02 0.83	0.26 0.011	0.16 0.128	0.20 0.051	0.07 0.492	1.00		
Hg	−0.08 0.473	−0.06 0.602	0.13 0.228	−0.26 0.014	−0.16 0.134	−0.10 0.348	−0.17 0.119	−0.21 0.047	−0.15 0.162	−0.19 0.081	−0.42 <0.001	1.00	
Pb	−0.21 0.043	−0.03 0.750	−0.13 0.233	0.145 0.171	0.28 0.008	0.14 0.200	0.21 0.042	0.18 0.090	0.29 0.005	0.16 0.119	0.80 <0.001	−0.31 0.003	1.00

## Data Availability

The data presented in this study are available upon request.
